# Innovative Tele-Instruction Approach Impacts Basic Life Support Performance: A Non-inferiority Trial

**DOI:** 10.3389/fmed.2022.825823

**Published:** 2022-05-12

**Authors:** Michael Tobias Schauwinhold, Michelle Schmidt, Jenny W. Rudolph, Martin Klasen, Sophie Isabelle Lambert, Alexander Krusch, Lina Vogt, Saša Sopka

**Affiliations:** ^1^AIXTRA—Competence Center for Training and Patient Safety, Medical Faculty, RWTH Aachen University, Aachen, Germany; ^2^Department of Anaesthesiology, University Hospital RWTH Aachen, Medical Faculty, RWTH Aachen University, Aachen, Germany; ^3^Center for Medical Simulation, Boston, MA, United States; ^4^Department of Anaesthesiology, Critical Care and Pain Medicine, Massachusetts General Hospital, Harvard Medical School, Boston, MA, United States

**Keywords:** basic life support (BLS), Cardiopulmonary Resuscitation (CPR), external chest compression (ECC), tele-instructor, telehealth, historical control, peer-teaching, non-inferiority

## Abstract

**Background:**

Sustaining Basic Life Support (BLS) training during the COVID-19 pandemic bears substantial challenges. The limited availability of highly qualified instructors and tight economic conditions complicates the delivery of these life-saving trainings. Consequently, innovative and resource-efficient approaches are needed to minimize or eliminate contagion while maintaining high training standards and managing learner anxiety related to infection risk.

**Methods:**

In a non-inferiority trial 346 first-year medical, dentistry, and physiotherapy students underwent BLS training at AIXTRA—Competence Center for Training and Patient Safety at the University Hospital RWTH Aachen. Our objectives were (1) to examine whether peer feedback BLS training supported by tele-instructors matches the learning performance of standard instructor-guided BLS training for laypersons; and (2) to minimize infection risk during BLS training. Therefore, in a parallel group design, we compared arm (1) Standard Instructor Feedback (SIF) BLS training (Historical control group of 2019) with arm (2) a Tele-Instructor Supported Peer-Feedback (TPF) BLS training (Intervention group of 2020). Both study arms were based on Peyton's 4-step approach. Before and after each training session, objective data for BLS performance (compression depth and rate) were recorded using a resuscitation manikin. We also assessed overall BLS performance via standardized instructor evaluation and student self-reports of confidence via questionnaire. Non-inferiority margins for the outcome parameters and sample size calculation were based on previous studies with SIF. Two-sided 95% confidence intervals were employed to determine significance of non-inferiority.

**Results:**

The results confirmed non-inferiority of TPF to SIF for all tested outcome parameters. A follow-up after 2 weeks found no confirmed COVID-19 infections among the participants.

**Conclusion:**

Tele-instructor supported peer feedback is a powerful alternative to in-person instructor feedback on BLS skills during a pandemic, where infection risk needs to be minimized while maximizing the quality of BLS skill learning.

**Trial registration:**

https://www.drks.de/drks_web/navigate.do?navigationId=trial.HTML&TRIAL_ID=DRKS00025199, Trial ID: DRKS00025199.

## Introduction

The COVID-19 pandemic poses challenges for nearly every aspect of healthcare and healthcare administration. Many of these challenges and risks can be mitigated by pausing or delaying certain kinds of care and encounters. Unfortunately, heart disease and cardiac arrest can neither be paused nor delayed. Sudden cardiac death remains the “top killer,” accounting for almost 9 million fatalities worldwide per year (1, 2). Thus, the need for emergency cardiac care remains constant. Starting CPR promptly with efficient BLS measures is the most important action before professional help arrives (3). Practical BLS training is therefore indispensable to qualify first responders as well as professionals (4–6). But how to prepare laypeople and new healthcare trainees to be ready with key skills, while mitigating infection risk and managing infection-related anxiety is a challenge.

BLS trainings are usually led by a trained instructor (7) and provided in an in-person setting. However, in a pandemic, clusters of people pose significant risk of virus transmission, accelerating the rate of new infections and resulting in a rising number of fatalities (8, 9). Reducing the number of participants trainings becomes extremely expensive making the spatial infrastructural requirements of practical training unattainable. Further, the number of available instructors might be reduced due to illness or quarantine requirements. Therefore, self-directed or peer-guided training approaches are of increasing interest when instructor-based trainings are not permitted or not feasible (10).

A pandemic requires inventive approaches to teaching and learning essential life-saving BLS skills to ensure personal protection and infection control (11, 12). The confluence of pressures to reduce expert input and test alternative peer-guided learning models with the imperatives of infection control during the pandemic urged us to evaluate a new approach. We reasoned that peer feedback aided by tele-instructor input would make it possible to reduce learner group size and to maintain physical distancing as well as infection control during the pandemic.

Peer-guided learning with peer feedback as a key component, is a promising technique in qualifying medical professionals and recommended by the 2020 American Heart Association Guidelines for Cardiopulmonary Resuscitation and Emergency Cardiovascular Care (13). A number of studies have shown positive effects of peer-guided learning for various teaching applications (14–17). It offers great potential to reduce the use of highly trained medical experts as instructors and to replace them completely or partially with peers (10, 17, 18). However, it is essential to assure that peer feedback is directed by the same clear and rigorous standards and instructions (19). We hypothesize that this can be addressed adequately by including a checklist.

Besides, we argue that it is beneficial to include a tele-instructor who can be consulted on demand and trouble-shoot the peer feedback. Remote guidance from a tele-instructor allows expert input, consults, and guidance to play a role in improving or adding to peer guidance. There are a number of examples where telemedicine approaches or telemedicine consultation of experts have been applied very successfully (20–23). This is particularly the case for the cross-location solution of clinical problems or feedback in decision support where the complexity is even higher (20, 21, 24).

Thus, our aim was to develop a tele-instructor supported peer feedback approach for BLS training and to test it for effectiveness with respect to essential learning outcomes. Even after the pandemic, this will ensure that efficient BLS training with comparable training results can be achieved in the absence of medical experts.

## Methods

### Ethics

Ethical approval (Ethical Committee 376/19 and 428/20) was provided 22.11.2019 and 12.11.2020 by the Ethical Committee of the University Hospital, RWTH Aachen, Pauwelsstraße 30, 52074 Aachen, Germany (Chairperson Prof. Dr. med. G. Schmalzing) and designed according to the ethical principles of the World Medical Association's Declaration of Helsinki (25).

### Participants

Participants were students during the first 2 weeks of their medical, dental, or physiotherapy studies at the RWTH Aachen University, Germany. Data assessment took place during a mandatory introductory course on emergency medicine in October 2019 (historical control group) and November 2020 (intervention group). Written informed consent was obtained from all participants.

### Study Design

We conducted an intervention study with a historical control group comparing two training methods for BLS skills. In detail, a well-established Standard Instructor Feedback (SIF) conducted by expert instructors (historical control group) was compared with an innovative composed Tele-Instructor Peer Feedback (TPF; intervention group). The TPF was based on peer-feedback, a checklist and optional self-created video recordings combined with an expert tele-instructor. As primary outcome parameters compression depth (CD) and compression rate (CR) within the BLS algorithm were investigated. Adherence to a BLS Algorithm (26, 27) and the assessed self-confidence of the learners were defined as secondary outcome parameters. In 2020, in acknowledgment of the increased risk of virus transmission via mouth-to-mouth ventilation, we omitted ventilations in accordance with the guidelines (26).

The trial aims to assess whether tele-instructor based BLS training was non-inferior to a conventional training approach (28). However, due to an increased infection risk in the 2020 COVID-19 pandemic, a control group using the conventional training approach with group sizes of 12 trainees was not possible. These circumstances led us to using a historical control group for the present study. To minimize any possible bias arising from the time lag, we selected a data set acquired with the conventional approach in the standard group size immediately before the pandemic (October 2019) as control group.

#### 2019 Data Set

The 2019 data set was part of a larger study comparing conventional training to a video-instructed peer-feedback (VPF) training without tele-instructor in a parallel group design. Since it was of no relevance for the topic under investigation, the VPF study arm was not considered in the present study. Results from this condition will be reported elsewhere. No data or results from the 2019 data set have been published before.

#### 2020 Data Set

Similarly, data acquisition in the 2020 study took place in two study arms. Since including an adequate control condition was not possible in 2020, we decided to collect data from a second intervention group, i.e., conventional training with a smaller group size (4 trainees), which can be equally compared with the historical control from 2019. Since this comparison was of no interest for the study presented here, it will be reported elsewhere.

**Figure 1:**
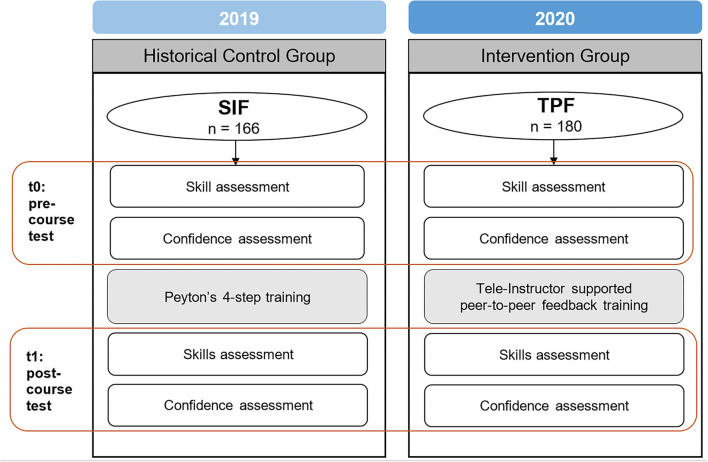
Flow Chart.

In both years, participants were randomly assigned to one of the two respective study arms (2019: video-instruction based training vs. conventional training in groups of twelve; 2020: tele-instructor-based training vs. conventional training in groups of four). A flow chart of this study is shown in Figure 1. To assure comparability of the training methods regarding complexity and time spent for training, study arms from both years followed a similar structure, based on Peyton's 4-step approach (29). Both training methods used a *Resusci Anne*^*TM*^ manikin (Laerdal, Stavanger, Norway). This widely used approach for teaching practical skills like BLS is based on Albert Bandura's social learning theory that emphasizes the social facets of observation, imitation, and modeling (30).

#### Arm 1: Standard Instructor Feedback Practical Training (SIF) (In-person Groups of 12 Learners From 2019 Data Set)

Following the Peyton four-step model, in step 1, a trained instructor demonstrated correct BLS performance without commentary. In step 2, the demonstration was enriched with detailed step-by-step explanation deconstructing individual steps. In step 3, instructor and student switched roles. The instructor performed BLS guided by the students explaining the steps. In step 4, all learners performed BLS on the manikin themselves and received individual feedback from a qualified instructor. Each participant went through at least two rounds à 2 min of CPR (total duration: 90 min). Vicarious learning strengthens the process as the participants observed the other learners while performing BLS and receiving feedback.

#### Arm 2: Tele-Instructor Guided Practical Training (TPF) (In-person Groups of 4 Learners From 2020 Data Set)

Following a modified Peyton four-step approach, in step 1, participants watched a video showing a trained instructor demonstrating correct BLS performance without commentary. In step 2, participants watched a video showing a trained instructor demonstrating correct BLS performance with detailed step-by-step explanations. Step 3 was omitted. In step 4, all teams of four students trained independently using the two manikins, assisted by a checklist of quality characteristics. A sample video of an ideal BLS performance was provided for reference and comparison to one's own performance. Subsequently, the peers were allowed to film each other during BLS performance in order to analyse videos of their own performance using the quality checklist. This allowed for both direct and vicarious learning about BLS performance. These activities were repeated until all students got the subjective impression that they had reached an acceptable level of performance and confidence (total duration: 45 min).

### Research Questions

**RQ1 (Primary research question):** Is TPF non-inferior to SIF with respect to BLS performance (CD and CR)?

**RQ2:** Is TPF non-inferior to SIF with respect to performance of the entire BLS algorithm?

**RQ3:** Is TPF non-inferior to SIF with respect to self-reported confidence concerning BLS skills?

### Skill Assessment

BLS performance was assessed before (t0) and after (t1) the training using the same Resusci Anne^TM^ (Laerdal, Stavanger, Norway) manikin. Students were instructed to imagine the manikin to be a person collapsing next to them and to take all required actions. The scenario was ended 120 s after the first chest compression. Compression depth (CD) and compression rate (CR) were recorded by the manikin's Laerdal PC SkillReporting System Software (Version 2.4.1, Laerdal, Stavanger, Norway).

Correct CD and CR after BLS training were the main learning outcomes. Based on the American Heart Association (AHA) guidelines (31), correct CD was defined as an average value between 50 and 59 mm (32–35). Correct CR was defined as an average rate of 100-120 compressions per minute (32, 36–38).

Participants' performance regarding the BLS algorithm was assessed by an expert rater *via* a standardized checklist covering safe approach, control of consciousness and of breathing, and emergency call. Sufficient adherence to the algorithm was approved if a participant performed more than 60% of the modified BLS-Algorithm adapted to the COVID-19 Pandemic (26) relevant items correctly.

Afterwards the participants rated their confidence (a) during CPR, (b) mastering an emergency situation and (c) applying BLS in a real-life situation with a non-responsive person. Ratings were obtained before and after training on a 6-point Likert scale (1 = “not at all confident,” 6 = “very confident”).

A graphical representation of the study design is depicted in Figure 1.

### Definition of Non-inferiority Margins

The definition of non-inferiority margins was based on the results of previous studies at our training center (39, 40) quantifying the rates of successful BLS after training with Peyton's 4-step approach for various samples of BLS-naive subjects. The results from these studies covered a range of 19% points for both CD (45-64%) and CR (33-52%). Since this outcome variation was present with the standard approach, any outcomes of another training method within these ranges were considered as non-inferior. Thus, for the comparison of TPF and SIF, Δ = −19% was defined as non-inferiority margins for both CD and CR.

Non-inferiority margins for BLS performance were also based on the aforementioned studies, showing a range of 27 percentage points (65-92%). Therefore, Δ = −27% was defined as the non-inferiority margin of the BLS performance.

Since there was no comparable data available for confidence ratings, a difference of −0.5 points (~8%) on the six-point Likert scale was defined as the non-inferiority margin.

### Sample Size Planning

Sample size planning for non-inferiority testing was performed in accordance with Blackwelder (41) with the Sealed Envelope Power Calculator (42). For an α significance level of.05 and a power (1 – β) of 90%, the required sample size was *N* = 236 for CD and *N* = 234 for CR. We decided for the larger sample size of *N* = 236 (118 per group).

### Randomization

Before the study was conducted, students were allocated to groups of 12 (2019) or 4 (2020) persons by an independent administrative employee of the student's deanery, who was blinded to the study. Allocation was stratified by gender and age to create homogenous groups using a sequence of random numbers. In a next step, groups were assigned to the study arms using a sequence of random numbers taking personnel resources as well as the facilities room and manikin options into account.

### Statistical Analysis

Data were analyzed with IBM SPSS Statistics Version 25 (IBM Corp., Armonk, NY, USA). Non-inferiority was assessed by comparing the percentage of successful performances (for CD and CR) after training in both study arms. We used two-sided 95% confidence intervals (CI, according to the recommendations of the CONSORT statement (43) to determine significance of non-inferiority. Significance of results was given for 95% CIs of empirical percentage differences excluding the non-inferiority margin values. CIs for the difference between percentages were calculated with the Wilson score interval method (44) for independent proportions. Analogously, the 95% CI of the difference between the Likert scale confidence ratings in both study arms was used.

### Infection Control and Safety Precautions

Intending to minimize the infection risk, each group of students started their trainings with a time delay of 15 min. The wearing of face masks, compliance with distance regulations, and regular use of hand disinfection was compulsory. Upon entering the training center, the body temperature of each student was measured using a contactless infrared thermometer. Cut off for exclusion was >37.5°C. A questionnaire on the current state of health completed the assessment. The code of behavior in the training center was provided online *via* a learning management system and refreshed at the beginning of each training. To reduce the likelihood of encounters between persons, there was one ascending and one descending staircase, and all walking ways on each floor of the training center were strictly one-way. All materials, chairs, tables, and door handles were disinfected after use at the end of each session. Hand sanitizer was available in every training room and lavatory. Signs repeating all instructions were mounted at the entry to all staircases and on the floor, signs explaining proper use of hand sanitizer were attached next to every disinfectant dispenser. This bundle of measures was approved by the occupational safety department of RWTH Aachen University Hospital and the crisis management team of RWTH Aachen University. Prophylactic PCR testing for the SARS CoV-2 virus was not possible due to capacity and time constraints. Corona rapid tests were not available in sufficient numbers in Germany at the time of data collection or too expensive. In addition, the significance back then was estimated being too low. Two weeks after the training we assessed symptoms of respiratory infection and COVID-19 test results as follow up of the effectiveness of our measures. The following characteristic COVID-19 symptoms were assessed: shortness of breath, coughing, sore throat, limb pain, general feeling of illness, and altered sense of taste or smell.

### Technical Requirements for the Peer-Feedback Arm

While no media support was required for the SIF method, in TPF group we used one notebook ensuring the tele-support and two tablets providing the instructional videos. Mobile as well as static solutions are possible.

## Results

### Sample Characteristics

Data from *N* = 395 participants (268 female, age 20.56 ± 3.68 years) were gathered. Due to absence during data collection, incomplete data or non-matching participant codes, 49 participants were excluded from the analyses leaving a final study sample of *n* = 346 (*n* = 180 in TPF; *n* = 166 SIF). Both study groups—SIF (2019) and TPF (2020)—were highly similar with respect to age (2019: 20.59 ± 3.64 years; 2020: 20.28 ± 2.79 years) and gender distribution (2019: 120 female, 55 male; 2020: 122 female, 57 male). Skills in resuscitation prior to participation in the study can be assumed for a part of the sample due to previous medical qualifications (2019: 17.6%; 2020: 23.3%), two-day courses in first aid (2019: 45.5%; 2020: 44.0% and 1-day courses in life-saving emergency measures (2019: 78.4%; 2020: 88.5%).

### Descriptive Data

Pre-training performance data and self-reported confidence ratings for the SIF group and the TPF group are reported in Table 1.

**Table 1:** Descriptive performance and target achievement data before (t0) and after the training.

	**SIF**		**TPF**	
	**M**	**SD**	**M**	**SD**
**t0**				
Average compression depth (mm)	40.86	18.02	44.19	13.20
Average compression rate (1/min)	88.39	37.40	100.74	26.83
	**Achieved**	***n***	**Achieved**	***n***
Correct compression depth (Total/%)	49 (35.5)	138	61 (33.9)	180
Correct compression rate (Total/%)	20 (11.6)	138	67 (37.2)	180
Correct BLS performance (Total/%)	77 (46.4)	166	76 (42.2)	180
**t1**				
Average compression depth (mm)	48.33	9.74	49.23	9.17
Average compression rate (1/min)	107.12	15.07	112.02	15.27
Confidence for CPR performance	5.10	0.78	5.08	0.78
Confidence for emergency situation	4.72	0.86	4.66	0.84
Confidence for real-life situation	4.86	1.11	5.10	0.87
	**Achieved**	***n***	**Achieved**	***n***
Correct compression depth (Total/%)	58 (41.4)	140	90 (51.1)	176
Correct compression rate (Total/%)	30 (21.4)	140	83 (47.2)	176
Correct BLS performance (Total/%)	145 (94.2)	154	149 (84.7)	176

*t0, Pre-course test; t1, Post-course test; SIF, Standard Instructor Feedback; TPF, Tele-Instructor Peer Feedback*.

### Non-inferiority Analysis

**Figure 2:**
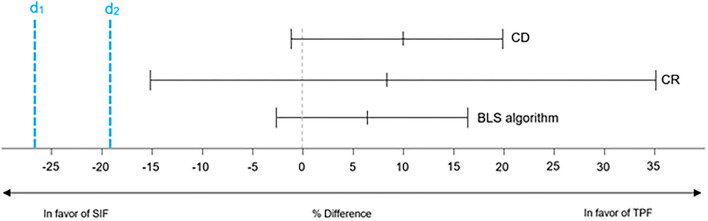
Non-Inferiority results for compression depth, compression rate and BLS algorithm. d1, non-inferiority margin set at Δ = −19% for compression depth and compression rate; d2, non-inferiority margin set at Δ = −27 % for BLS algorithm; CD, Compression depth; CR, Compression rate; BLS, Basic Life Support; SIF, Standard Instructor Feedback; TPF, Tele-Instructor Peer Feedback.

**Figure 3:**
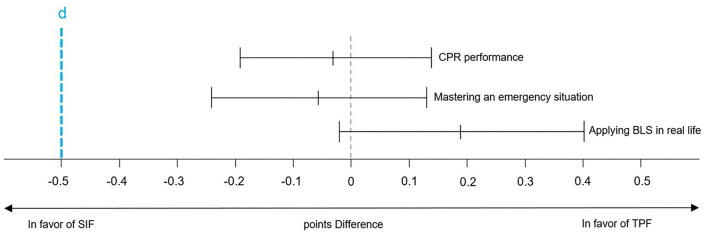
Non-inferiority confidence ratings. CPR, Cardiopulmonary Resuscitation; BLS, Basic Life Support; SIF, Standard Instructor Feedback; TPF, Tele-Instructor Peer Feedback.

Results of non-inferiority analyses are displayed in Figures 2, 3 which show the respective proportion differences between the SIF group of 2019 and the TPF group of 2020 on a 95% CI. Values < 0 favor SIF and values > 0 favor TPF. The blue line indicates the respective non-inferiority margin (Δ). For CD and CR, the inferiority margin was set at Δ = −19 % whereas for BLS performance data it was set at Δ = −27 % based on empirical values from prior data. The non-inferiority margin for confidence ratings was Δ = −0.5 points.

#### Compression Depth

After training, 41.4 % of the participants in the SIF and 51.1 % in the TPF group achieved a correct CD, resulting in a proportion difference of 9.7 % in favor of TPF. The 95% CI for the proportion difference was −1.4-20.4%. The results indicate significant non-inferiority of the TPF group.

#### Compression Rate

After training, 21.4% of the participants in the SIF and 47.2% in the TPF group achieved a correct CR, resulting in a proportion difference of 25.8% in favor of TPF. The 95% CI for the proportion difference was 15.3-35.2%. The results indicate a significant non-inferiority of the TPF group.

#### BLS Performance

After training, 94.2% of the participants in the SIF and 84.7% in the TPF group achieved a correct CD, resulting in a proportion difference of 9.5% in favor of SIF. The 95% CI for the proportion difference was 2.8-16.2%. The results indicate significant non-inferiority of the TPF group.

#### Confidence Ratings

Mean differences between the two groups were 0.02 points (95 % CI: −0.14-0.19) regarding confidence in one's CPR performance, 0.06 points (95 % CI: −0.12-0.24) in terms of confidence in an emergency situation and 0.24 points (95 % CI: −0.40-0.23) in real-life situations. The results indicate significant non-inferiority of the TPF group for all three items. The results are depicted in Table 1 and Figure 3.

### COVID-19 Health Measurements

#### Body Temperature

Average body temperature on the day of study participation was 35,99°C (SD: 0,69°C). One participant was excluded from the training due to high temperature (37,8°C).

#### Infection Risk

After 2 weeks, 243 participants responded to the follow-up questionnaire. Among these, 236 participants (97.1%) reported none of the assessed COVID-19 symptoms. No participant reported a positive PCR test. Among the remaining 7 participants, 2 reported coughing, 4 reported a sore throat, one reported shortness of breath, and one reported general feeling of illness. In summary, there was no indication for an increased prevalence of COVID-19 cases after training participation.

### Tele-Instructor Peer Feedback Efficiency

It is important to highlight that the TPF group needed only half of the training time of SIF to accomplish comparable results. The essential instructors could offer support to the double number of participants. This does not take into account the savings made by halving the training time for the participants. From an economic point of view, personnel cost thus could be significantly reduced using TPF instead of SIF.

## Discussion

In times of a pandemic, when physical distancing is one of the most important methods of infection control, how to both train large numbers of people and do it safely require innovation in training methods. In the present study, we investigated non-inferiority of a new pandemic-adapted learning approach addressing BLS, with respect to essential learning outcomes. The results confirmed non-inferiority of the tele-instructor guided peer feedback method with respect to compression depth and compression rate.

Furthermore, the overall performance of the BLS algorithm, and self-reported confidence were comparable between groups as well. Results of the health status follow-up showed no sign of an elevated COVID-19 infection risk for the BLS course participants. Thus, BLS training with tele-instructor guided peer feedback is a valuable and effective alternative to traditional large group in-person instructor training. It combines the spatial and temporal flexibility of peer teaching with the expert support of instructor guidance, while simultaneously allowing for small group sizes and low costs. This seems an optimal way to fight sudden cardiac death even in pandemic times.

Beyond aspects of practical realization, the use of peer feedback in medical education has long since been discussed and recommended (45). Nonetheless, it is not well anchored as a teaching method in current medical curricula. One reason could be a potential pitfall of peer teaching: peers are usually no experts, and feedback may sometimes be erroneous. Accordingly, it seems that some clinicians and medical educators do not trust the method and avoid it in their teaching (46). TPF can address this issue in a two-fold manner. First, the use of a checklist makes peer feedback and its underlying criteria more objective. Second, the consultation of a professional tele-instructor adds essential expert knowledge and can avoid errors and uncertainties. The TPF approach accordingly combines positive learning effects of peer feedback and instructor guidance. We therefore suggest that TPF can enhance clinical educator's trust in peer feedback and foster the implementation of peer feedback teaching in the medical curriculum.

Both interventions differed in a number of factors which may have contributed to the comparable training effects to varying degrees. A potential factor to students' non-inferior performance of CD and CR in the TPF group could be the possibility to analyse their own performance in detail by means of the video recordings. This hypothesis is in line with the findings by Bezemer et al. who found beneficial effects of video recordings on subsequent performance and team communication on the surgical ward (47). To continue, the amount of practice time on the manikin is likely to have differed among groups. Whereas the degree of peer observation was higher among participants in the SIF condition (12 participants and 1 manikin), the ratio of manikin to student in the TPF group was 1:2 and thus allowed for more practice-oriented experience. Referring to Bandura's Social Learning Theory (30), learning in the SIF notably occurred through observation of the instructor and peers, whereas learning in the TPF group was particularly enhanced by the practical reproduction of the acquired skill.

Amparore et al. exemplified the decline in continuing medical education during the pandemic in urology residents (48). Due to its low requirements, the TPF approach could be used for BLS trainings in other settings like universities, schools, sports clubs, companies etc. as well. Moreover, the teaching approach tested is not limited to BLS training. By supplying learners with excellent educational material in advance, practical training of almost every basic as well as some complex clinical skills (e.g., central venous catheters or airway management techniques) can be trained using a tele-instructor. Even continuing medical education in practical skills could be provided wider by means of TPF and thus be strengthened.

The necessary technical equipment can be acquired at low costs and easily adjusted to various settings in order to vastly reduce the deployment of instructors. Necessary skills using videoconferencing software (e.g., managing breakout sessions, using the chat) are easily acquired. Furthermore, the online support of trainees can be performed in home office. During a pandemic this can be an enormous advantage regarding the feasibility of BLS trainings. In addition to a minimized infection risk, instructors can back up each other, which remains relevant even after the pandemic. In our current situation, distance regulations required 4 m^2^ space per participant. Hand disinfection needed to be available in every room and lavatories had to be close by. An optimal floor plan is therefore required to ensure participants can enter and leave rooms without queuing and passing by other participants.

The present study has some limitations. Our study sample consisted of young, well-educated, and medically interested learners. Studies on comparable samples are widely distributed, and a potential transferability to the general population has been claimed as the participants had not yet entered the regular medical curriculum (49). Nevertheless, generalization of our findings requires investigations of other layperson populations. This could be achieved with a superiority trial based on an intention to treat sample in the context of public courses on resuscitation. Taking into account that the strength of TPF lies in its broad and easy applicability, this design might prove that TPF—on cohort level—achieves even better training results than SIF since it can reach more trainees. This is especially of interest in times of a pandemic. However, easier applicability may also promote population-wide BLS trainings beyond crisis situations.

Moreover, the TPF group could decide for themselves when to contact the tele-instructor, which bears the risk of undetected inaccurate performance. To reduce this threat, either contacting the tele-instructor could be mandatory or the training room could be under video surveillance while training takes place, empowering the tele-instructor to intervene if there is a need for improvement. The second option will make strict rules for privacy protection necessary.

Teaching the COVID-algorithm of BLS (26), this study does not permit any conclusion on rescue breathing. Acknowledging the possibility for untrained laypersons to perform compression-only CPR (31), this aspect may be considered negligible. However, as soon as it is reasonably possible to practice rescue breathing without elevated risk of infection, this aspect should also be examined in further investigations. Knowing that bag-mask ventilation with an HME filter could be an alternative to mouth-to-mouth ventilation for rescuers, we decided not to use this technique acknowledging of the lack of necessary equipment for laypersons.

## Data Availability Statement

The raw data supporting the conclusions of this article will be made available by the authors, without undue reservation.

## Ethics Statement

The studies involving human participants were reviewed and approved by Ethical Committee of the University Hospital Chairperson Prof. Dr. med. G. Schmalzing RWTH Aachen Pauwelsstraße 30 52074 Aachen Germany. Written informed consent was obtained from all participants.

## Author Contributions

MTS contributed substantially to the designing of this study as well as the acquisition, analysis, and interpretation of the data and has written the manuscript. MS, MK, and SS contributed substantially to the planning and designing of this study, the statistical analysis as well as the interpretation of the data. JR critically revised the manuscript for important intellectual content and made substantial contributions to the manuscript. SL, LV, and SS assisted in critically reviewing the manuscript. AK substantially contributed to the practical implementation of the study. SS supervised the study and supported MTS as senior investigator. All authors have made contributions to the manuscript and reviewed and revised the manuscript.

## Funding

This study was financed by regular departmental funding for research and education of RWTH Aachen.

## Conflict of Interest

The authors declare that the research was conducted in the absence of any commercial or financial relationships that could be construed as a potential conflict of interest.

## Publisher's Note

All claims expressed in this article are solely those of the authors and do not necessarily represent those of their affiliated organizations, or those of the publisher, the editors and the reviewers. Any product that may be evaluated in this article, or claim that may be made by its manufacturer, is not guaranteed or endorsed by the publisher.

## Abbreviations

BLS: Basic Life Support
CD: Compression Depth
CI: Confidence Interval
CPR: Cardiopulmonary Resuscitation
CR: Compression Rate
ECC: External Chest Compression
SIF: Standard Instructor Feedback
TPF: Tele-Instructor Peer-Feedback.
