# Supplementary material for: Innovative Tele-Instruction Approach Impacts Basic Life Support Performance: A Non-inferiority Trial
Source: Front Med (Lausanne). 2022 May 12;9:825823. doi: 10.3389/fmed.2022.825823 (PMC9134732; doi:10.3389/fmed.2022.825823)
Supplement: Supplementary file 1 [file Data_Sheet_1.pdf]

CONSORT Statement 2006 - Checklist for Non-inferiority and Equivalence Trials

Items to include when reporting a non-inferiority or equivalence randomized trial

PAPER SECTION **And topic**	**Item**	**Descriptor**	**Reported on Page #**
TITLE & ABSTRACT	1	How participants were allocated to interventions (*e.g.*, "random allocation", "randomized", or "randomly assigned"), *specifying that the trial is a non-inferiority or equivalence trial.*	1-2
INTRODUCTION Background	2	Scientific background and explanation of rationale, *including the rationale for using a non-inferiority or equivalence design.*	2
METHODS Participants	3	Eligibility criteria for participants *(detailing whether participants in the non-inferiority or equivalence trial are similar to those in any trial(s) that established efficacy of the reference treatment)* and the settings and locations where the data were collected.	2
Interventions	4	Precise details of the interventions intended for each group *detailing whether the reference treatment in the non-inferiority or equivalence trial is identical (or very similar) to that in any trial(s) that established efficacy,* and how and when they were actually administered.	2-4
Objectives	5	Specific objectives and hypotheses, *including the hypothesis concerning non-inferiority or equivalence.*	4
Outcomes	6	Clearly defined primary and secondary outcome measures *detailing whether the outcomes in the non-inferiority or equivalence trial are identical (or very similar) to those in any trial(s) that established efficacy of the reference treatment* and, when applicable, any methods used to enhance the quality of measurements (*e.g.*, multiple observations, training of assessors).	4
Sample size	7	How sample size was determined *detailing whether it was calculated using a non-inferiority or equivalence criterion and specifying the margin of equivalence with the rationale for its choice.* When applicable, explanation of any interim analyses and stopping rules (*and whether related to a non-inferiority or equivalence hypothesis*).	4
Randomization -- Sequence generation	8	Method used to generate the random allocation sequence, including details of any restrictions (*e.g.*, blocking, stratification)	4
Randomization -- Allocation concealment	9	Method used to implement the random allocation sequence (*e.g.*, numbered containers or central telephone), clarifying whether the sequence was concealed until interventions were assigned.	4
Randomization -- Implementation	10	Who generated the allocation sequence, who enrolled participants, and who assigned participants to their groups.	4
Blinding (masking)	11	Whether or not participants, those administering the interventions, and those assessing the outcomes were blinded to group assignment. If done, how the success of blinding was evaluated.	4
Statistical methods	12	Statistical methods used to compare groups for primary outcome(s), *specifying whether a one or two-sided confidence interval approach was used.* Methods for additional analyses, such as subgroup analyses and adjusted analyses.	4-5
RESULTS Participant flow	13	Flow of participants through each stage (a diagram is strongly recommended). Specifically, for each group report the numbers of participants randomly assigned, receiving intended treatment, completing the study protocol, and analyzed for the primary outcome. Describe protocol deviations from study as planned, together with reasons.	3, 5
Recruitment	14	Dates defining the periods of recruitment and follow-up.	2
Baseline data	15	Baseline demographic and clinical characteristics of each group.	5
Numbers analyzed	16	Number of participants (denominator) in each group included in each analysis and whether the analysis was *"intention-to-treat" and/or alternative analyses were conducted.* State the results in absolute numbers when feasible (*e.g.*, 10/20, not 50%).	3, 5
Outcomes and	17	For each primary and secondary outcome, a summary of results	5-6

estimation		for each group, and the estimated effect size and its precision (*e.g.*, 95% confidence interval). *For the outcome(s) for which non-inferiority or equivalence is hypothesized, a figure showing confidence intervals and margins of equivalence may be useful.*	
Ancillary analyses	18	Address multiplicity by reporting any other analyses performed, including subgroup analyses and adjusted analyses, indicating those pre-specified and those exploratory.	-
Adverse events	19	All important adverse events or side effects in each intervention group.	-
DISCUSSION Interpretation	20	Interpretation of the results, taking into account the *non-inferiority or equivalence hypothesis and any other* study hypotheses, sources of potential bias or imprecision and the dangers associated with multiplicity of analyses and outcomes.	7-8
Generalizability	21	Generalizability (external validity) of the trial findings.	8
Overall evidence	22	General interpretation of the results in the context of current evidence.	7-8
