# Supplementary material for: Innovative Tele-Instruction Approach Impacts Basic Life Support Performance: A Non-inferiority Trial
Source: Front Med (Lausanne). 2022 May 12;9:825823. doi: 10.3389/fmed.2022.825823 (PMC9134732; doi:10.3389/fmed.2022.825823)
Supplement: Supplementary file 2 [file Data_Sheet_2.PDF]

Initial Assessment

Rating Checklist

Each item on the checklist is weighted with one point. A minimum of 0 and a maximum of 8 points can be achieved.

- ☐ Safe approach / self-protection
- ☐ Contacting and touching the patient
- ☐ Call for help
- ☐ Breath control (seeing)
- ☐ Breath control (>5sec and <10sec)
- ☐ Emergency call (112)
- ☐ Start chest compressions without delay
- ☐ Correct pressure point (centre of thorax)
