# Supplementary material for: Innovative Tele-Instruction Approach Impacts Basic Life Support Performance: A Non-inferiority Trial
Source: Front Med (Lausanne). 2022 May 12;9:825823. doi: 10.3389/fmed.2022.825823 (PMC9134732; doi:10.3389/fmed.2022.825823)
Supplement: Supplementary file 3 [file Data_Sheet_3.PDF]

Current state of health

Please answer the following questions honestly.

1) Have they been tested for SARS-CoV-II within the last 14 days?

o Yes o No

2) If the answer to question 1) is yes: Did they test positive for SARS-CoV-II?

o Yes o No

3) In the last 14 days, have you knowingly had contact with a person, who has been confirmed to be infected with the SARS-CoV-II virus, or are you in quarantine ordered by the authorities?

o Yes o No

4) Are you currently experiencing one or more of the following symptoms?
Please mark the options that apply. Multiple responses are possible.

o Shortness of breath
o Cough
o Cough
o Rhinitis
o Sore throat
o aching limbs
o general feeling of illness
o changes in odor/taste or loss
o I have not experienced any of the of the symptoms mentioned above.

o I have not experienced any of the of the symptoms mentioned above.

If you have checked an answer in the left column for any of the questions, please contact the student assistant supervising you or one of the medical faculty members directly.

Thank you!!
