# Supplementary material for: Innovative Tele-Instruction Approach Impacts Basic Life Support Performance: A Non-inferiority Trial
Source: Front Med (Lausanne). 2022 May 12;9:825823. doi: 10.3389/fmed.2022.825823 (PMC9134732; doi:10.3389/fmed.2022.825823)
Supplement: Supplementary file 4 [file Data_Sheet_4.PDF]

Checklist

- ❒ **Safe approach & self-protection**
  Includes a brief look over the overall situation for possible hazards (visible and invisible)

- ❒ **The patient is spoken to loudly and clearly**

- ❒ **The patient is touched**
  Brief tactile stimulus by touching.

- ❒ **"HELP!" is called.**

- ❒ **Breath control!**
  - ❒ **The duration of the control is 10 seconds.**
    (Seeing)
- ❒ **The patient's mouth and nose are covered.**

- ❒ **The emergency call 112 is made or initiated!**

- ❒ **The time until the start of chest compressions is as short as possible!**
  - ❒ **<u>Immediately</u> in the absence of breathing on undressed upper body**

- ❒ **Pressure point in the middle of the chest**

- ❒ **The depth of compressions is at least 5-6 cm over the entire period!**

- ❒ **The frequency of compressions is 100 - 120 per minute!**
  Frequency control possible with a watch, stopwatch or smartphone (120 per minute = compress 2 times every second).

- ❒ **After each compression, the chest is completely released!**

- ❒ **Position of the arms - stretched through when compressing**
